# Mass spectrometry based metabolomics approach on the elucidation of volatile metabolites formation in fermented foods: A mini review

**DOI:** 10.1007/s10068-021-00917-9

**Published:** 2021-07-08

**Authors:** Min Kyung Park, Young-Suk Kim

**Affiliations:** Department of Food Science and Engineering, Ewha Womans University, Seoul, 03760 Republic of Korea

**Keywords:** Metabolomics, Mass spectrometry, Fermentation, Volatile metabolites, Metabolic pathway

## Abstract

Metabolomics can be applied for comparative and quantitative analyses of the metabolic changes induced by microorganisms during fermentation. In particular, mass spectrometry (MS) is a powerful tool for metabolomics that is widely used for elucidating biomarkers and patterns of metabolic changes. Fermentation involves the production of volatile metabolites via diverse and complex metabolic pathways by the activities of microbial enzymes. These metabolites can greatly affect the organoleptic properties of fermented foods. This review provides an overview of the MS-based metabolomics techniques applied in studies of fermented foods, and the major metabolic pathways and metabolites (e.g., sugars, amino acids, and fatty acids) derived from their metabolism. In addition, we suggest an efficient tool for understanding the metabolic patterns and for identifying novel markers in fermented foods.

## Mass spectrometry-based metabolomics strategies

Omics tools can be used as a platform to provide strategies for controlling and changing the qualities of fermented foods. The various types of omics can be divided according to the target compounds, such as genes (genomics), mRNA (transcriptomics), proteins (proteomics), and metabolites (metabolomics). In particular, metabolomics is a comprehensive and quantitative approach for determining dynamic metabolic changes, being a powerful tool that can be efficiently applied in diverse scientific fields, such as food science, biochemistry, and drug discovery (Caboni et al., 2019; Migaud et al., 2020). Metabolites are low-molecular-weight organic compounds (< 1000 Da) in cellular systems that are related to nutritional values, sensory properties, and the generation of energy for survival. The most prevalent analytical tools for a metabolomics analysis are currently nuclear magnetic resonance (NMR) (Cho et al., 2007; Blakebrough-Hall et al., 2020) and chromatography-mass spectrometry (MS) (Gao and Xu, 2015). In particular, MS-based metabolomics analysis has been widely used due to its high selectivity and sensitivity, and potential in identifying metabolites (Dettmer et al., 2007). MS-based instrumental techniques including gas chromatography (GC)-MS, liquid chromatography (LC)-MS, and capillary electrophoresis (CE)-MS, are generally applied to investigate a large range of metabolites.

Table 1 lists the MS-based metabolomics that are actively used as tools for monitoring changes in intra- and extracellular metabolites during fermentation, for evaluating metabolic activities, and the non-target effects of mixed culture, for discriminating geographical origins, and for identifying biomarkers or key components related to functional and organoleptic properties. Alves et al. (2020) suggested how the fingerprinting of beer volatiles varied with the brewing steps. Lee et al. (2014) compared the primary and secondary metabolites profiles of *doenjang* during its industrial processing, including raw material (soybean), steaming step (1 day), drying (2 days), *meju* fermentation (3, 4, and 17 days), brining (22, 40, and 51 days), and aging (81 and 141 days). Metabolic data sets obtained from GC-time of flight (TOF)/MS and ultra-performance liquid chromatography (UPLC)-TOF/MS indicated the major metabolic changes associated with each process. A metabolomics approach can also be used evaluate the metabolic activity, which is related to the levels of specific metabolites produced. For example, knowledge of the profiles of metabolites produced during fermentation could be useful when attempting to enhance the functional properties of cheese. (Hagi et al., 2019). This could also provide some clues about the effects of mixed cultures, the discrimination of geographical origins, or finding novel biomarkers in fermented foods. Song et al. (2020) successfully determined the geographical origin of baijiu samples using volatile metabolite profiles, while Park and Kim (2020) revealed the microbial-specific metabolites in fermented soybeans using the profiles of primary and volatile metabolites and multivariate statistical analysis.

**Table 1:** Applications of metabolomics strategy

Application fields	Sample	Target compounds	Instrument	Refs^a^
Tracking of metabolic changes during fermentation	*Deonjang*	Primary metabolites	GC-TOF/MS	(Lee et al., 2014)
		Secondary metabolites	UPLC-Q-TOF/MS	
	*Beer*	Volatile metabolites	GC-MS	(Alves et al., 2020)
	*Black tea*	Secondary metabolites	HPLC-Q-TOF/MS	(Tan et al., 2016)
Metabolic activity evaluation	*Deonjang*	Volatile metabolites	GC-MS	(Kum et al., 2015)
		Enzymatic activities	Spectrometry	
	*Cheese*	Primary metabolites	HPLC	(Hagi et al., 2019)
		Secondary metabolites	LC–MS	
Non-target effects of mixed-culture Discrimination of origin	*Cheese*	Primary metabolites	UPLC-Q-TOF/MS	(Gu et al., 2020)
		Volatile metabolites	GC-MS	
	*Makgeolli*	Primary metabolites	GC-TOF/MS	(Son et al., 2018)
		Volatile metabolites	GC-MS	
	*Wine*	Volatile metabolites	GC-MS	(Englezos et al., 2018)
	*Deonjang*	Primary metabolites	GC-TOF/MS	(Lee et al., 2017)
		Secondary metabolites	LC-ESI/MS	
	*Soybeans*	Primary metabolites	GC-TOF/MS	(Lee et al., 2019)
		Secondary metabolites	LC-Orbitrap MS	
	*Baijiu*	Volatile metabolites	GC × GC-TOF/MS	(Song et al., 2020)
Finding novel biomarker	*Fermented soybeans*	Primary metabolites	GC-TOF/MS	(Park and Kim, 2020)
		Volatile metabolites	GC-MS	
	*Goat milk*	Primary metabolites	GC-MS	(Scano et al., 2014)

^a^Reference

Metabolomics approaches can be divided into two strategies, ‘targeted’ or ‘non-targeted’. Targeted approaches focus on the quantification and identification of preselected metabolites using references libraries (Gold et al., 2015). In targeted analysis, it can provide high selectivity and sensitivity, while it cannot obtain global analysis of metabolome, because it covers only certain metabolite-specific responses. In contrast to targeted metabolomics, non-targeted approaches deal with information on global metabolic changes and clues, possibly leading to the identification of novel biomarkers (Zhao et al., 2018). In non-target analysis, metabolites are not identified and the spectral data of all potential compounds are not preselected (Tikunov et al., 2005). In general, the data collected by non-target analysis follow four basic steps: deconvolution, alignment, filtering and gap filling (Mastrangelo et al., 2015). Before the identification of metabolites, all data sets need to be evaluated using multivariate statistical analysis. Thus, positive identification of metabolites is priority matter in non-target analysis, because enormous quantity of data and lack of information on data can limit the identification.

The success of an investigation of metabolomics is highly dependent on the overall experimental workflow, including sample collection and data interpretation. When analyzing a sample it is necessary to avoid changing the composition of the metabolites, such as via contamination. Extracting volatile metabolites is particularly difficult due to their sensitivity and instability, and this is performed using solvent-free methods [static and dynamic headspace extraction, solid-phase microextraction (SPME), and stir bar sorptive extraction (SBSE)] and solvent based extractions [simultaneous distillation extraction (SDE) and solvent-assisted flavor evaporation (SAFE)]. Solvent-based extraction can require further sample preparation, such as solid phase extraction (SPE), solvent evaporation, and chemical derivatization, in order to optimize them for instrumental analytical techniques.

Metabolomics data sets are commonly very large, and so diverse data-mining analysis techniques such as, principle component analysis (PCA), partial least squares (PLS), PLS- discriminant analysis (PLS-DA), and orthogonal projection to latent structures (OPLS), are often used to extract relevant information. Applying such statistical data-mining analysis methods in metabolomics research can identify treatment differences or patterns in large data sets. (Hendriks et al., 2011). They result in the projection of the original data on a lower dimensional space capturing as much as possible of the information in the data, that is, the observed variation (Saccenti et al., 2014).

Several software tools have recently become available for the functional and biological interpretation of metabolic data sets. Some tools can map and display associated metabolic data to provide visual representations of metabolism. (Chagoyen and Pazos, 2012). For example, kyoto encyclopedia of genes and genomes (KEGG)’s pathway browser (https://www.genome.jp/kegg) can provide a global view of the metabolism and a list of the involved pathways (Okuda et al., 2008). Also, MetaboAnalyst is a comprehensive Web-based tool that makes it easy to perform metabolomics data analysis, visualization, and functional interpretation. (Chong et al., 2018). In addition, the open-source R software contains a powerful set of functions for creating graphics, from fairly simple graphs using base graphics commands to highly sophisticated graphs using one of several advanced graphics packages. (Grace and Hudson, 2016). However, despite the many developed interpretation techniques, there remains a need to create new analytical platforms for interpreting metabolic data sets more efficiently and accurately.

## Fermentation, dynamic metabolic changes derived from microorganisms

Fermentation is a metabolic process associated with chemical break-down of macro-molecules (carbohydrates, proteins, and lipids) and the synthesis of micromolecules (e.g. volatile metabolites) by microorganisms. The microbial metabolic processes that occur during fermentation include a series of catabolism and anabolism steps involving the breaking down and synthesis of diverse metabolites. In the primary stage of fermentation, microorganisms grow rapidly on the surfaces of surrounding ingredients and usually make some metabolites associated with the growth phase, such as the synthesis of DNA, RNA, and vitamins, the degradation of macromolecular compounds, (carbohydrates, proteins, and lipids), and the generation of energy (ATP, NADH, and others) for their survival (Franco and Pérez‐Díaz, 2013). Various hydrolytic enzymes (e.g. amylase, glucoamylase, protease, and lipase) are also involved in the degradation of macromolecules to small molecules (sugars, amino acids, and fatty acids), which can be precursors for secondary metabolic reactions (Park et al., 2010). When microorganisms are present in the later exponential phase or stationary phase, they can form various secondary metabolites related to the functional or organoleptic properties of fermented foods. In particular, volatile metabolites comprise a chemically diverse group of organic compounds that generally have molecular weights in the range of 50–200 Daltons (Rowan, 2011). These compounds significantly affect the qualities of fermented foods, although they comprise only a small proportion of the total number of metabolites produced by microbes.

**Fig. 1:**
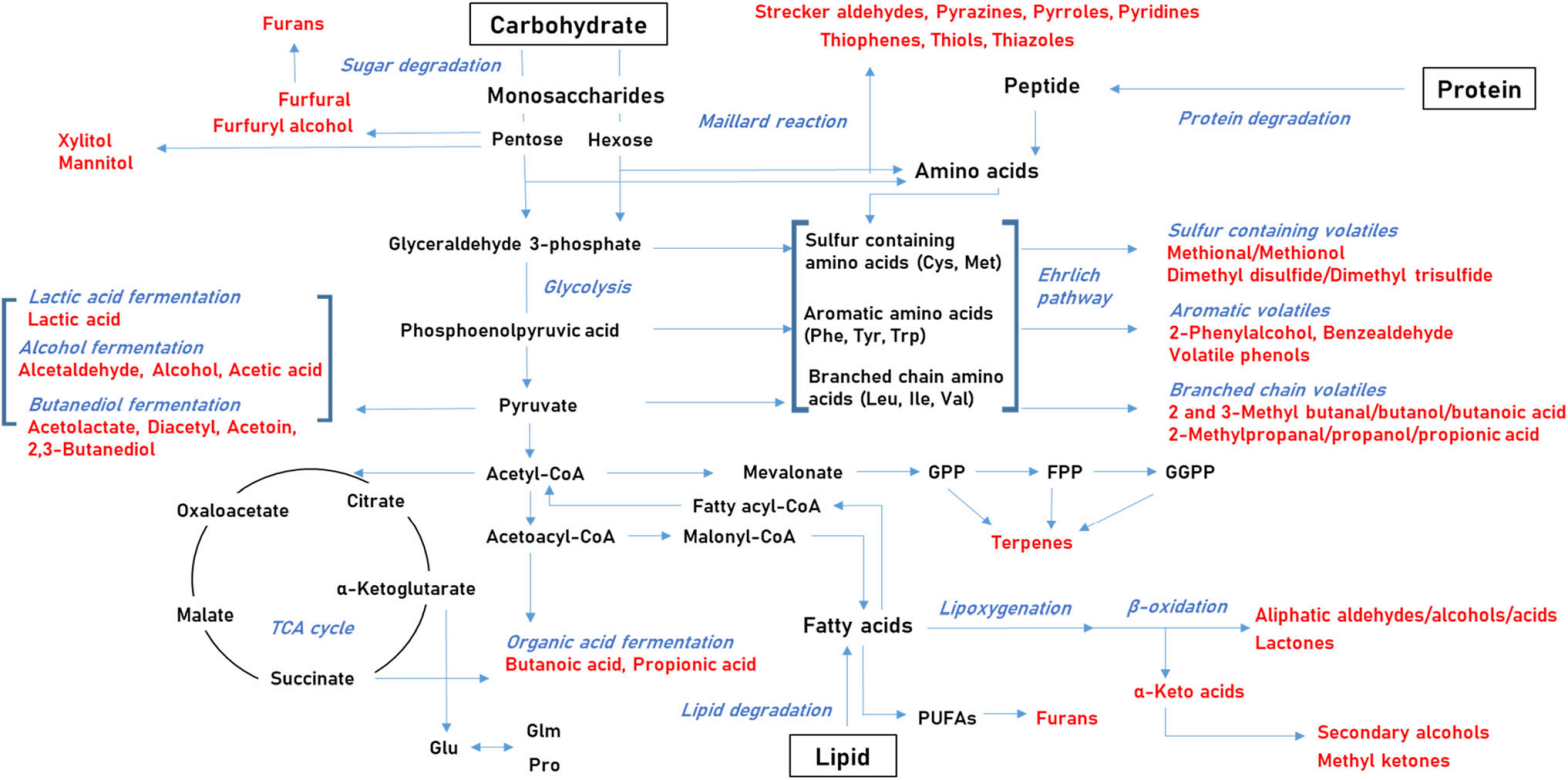
Main pathways for microorganisms to produce volatile metabolites. All abbreviations were shown as below; cysteine (Cys), methionine (Met), phenylalanine (Phe), tyrosine (Tyr), tryptophan (Trp), leucine (Leu), isoleucine (Ile), valine (Val), geranyl pyrophosphate (GPP), farnesyl pyrophosphate (FPP), geranylgeranyl pyrophosphate (GGPP), polyunsaturated fatty acids (PUFAs), glutamate (Glu), glutamine (Glm), and proline (Pro)

Based on relevant previous studies (Chen, 2008; Jo et al., 2011; Ferreira and Guido, 2018; Yu et al., 2014)**,** Fig. 1 presents the main microbial metabolic processes involved in the production of volatile metabolites during fermentation, including glycolysis, Ehrlich pathway (amino acid catabolism), lipoxygenation, oxidation/reduction. Multiple microbial metabolic are linked to each metabolite, but certain volatile metabolites can be generated only from specific ones. (Horak et al., 2019). For example, some intermediate metabolites such as pyruvate and acetyl-CoA are linked to several metabolic processes. Also, sulfur-containing compounds are specifically derived from sulfur-containing amino acids. The present study investigated fermentation processes by dividing metabolism into three parts involving carbohydrates, amino acids, and fatty acids.

**Fig. 2:**
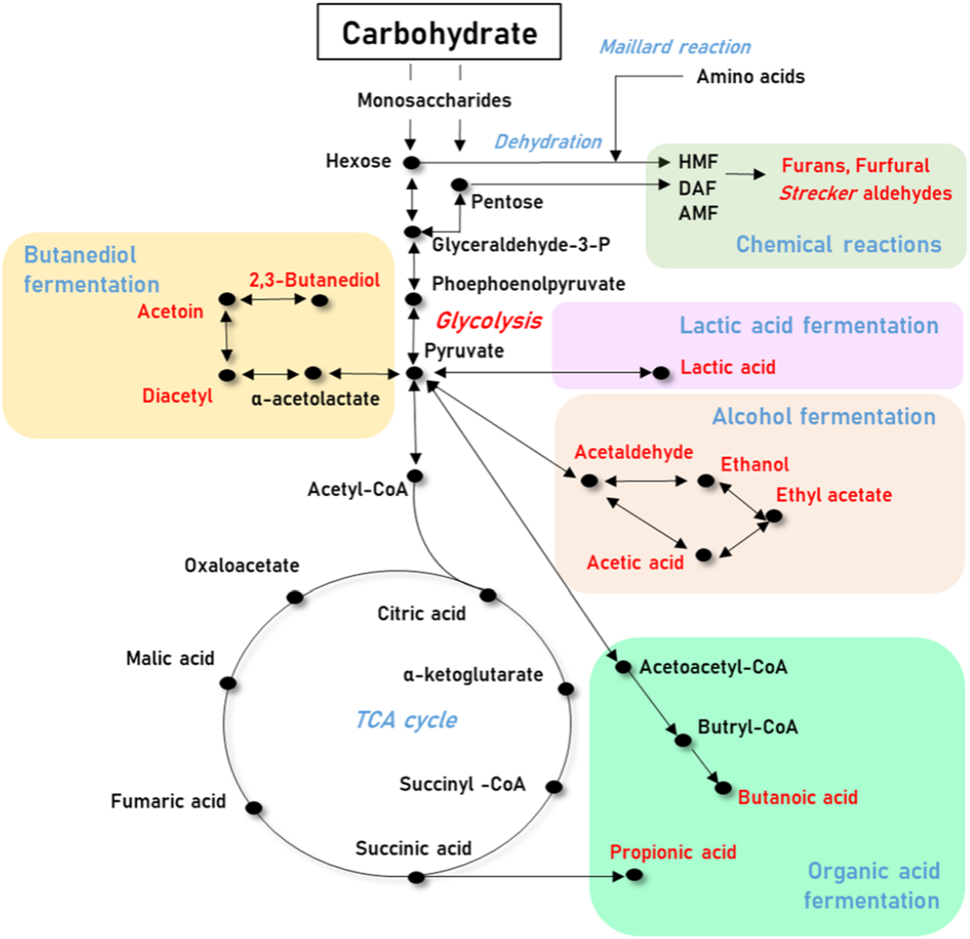
Overview of the main carbohydrate metabolism related to form volatile metabolites

Carbohydrate metabolism (Fig. 2) provides a carbon source for the formation of volatile metabolites as well as microorganism growth (Mao et al., 2005). It also produces other precursors needed for the formation of volatile metabolites, such as amino acids, fatty acids, and metabolic fuels (NADH, NAD^+^, ATP, and etc.) related to other metabolic pathways (Berg et al., 2002). Chemical and biological processes occur simultaneously during fermentation. In particular, the Maillard reaction is a common chemical reaction (non-enzymatic reaction) that occurs between amino acids and reducing sugars during heating. Amadori compounds are formed in the initial step of this reaction, which is followed by a series of rearrangements, dehydrations, and cyclization to produce volatiles such as furans, furfural, and Strecker aldehydes, as well as color in fermented foods. (Golon et al., 2014).

There are also several important biological pathways involved in the formation of volatile metabolites during the metabolism of carbohydrates, including the fermentation of lactic acid, alcohol, butanediol, and organic acids (butanoic acid and propanoic acid). Lactic acid fermentation is the simplest type of fermentation, which involves the production of lactic acid from pyruvate. Lactic acid can influence the flavor of fermented foods by the production of acids and the lowering of pH, which increases sourness (McFeeters, 2004). The pathways can be divided into the fermentation of homolactic acid (only producing lactic acid) and heterolactic acid fermentation (mainly producing lactic acid, also converting to ethanol and carbon dioxide), with lactic acid bacteria being mainly responsible (Kim et al., 2020). Alcoholic fermentation, in which the main products are ethanol and carbon dioxide, is commonly used for producing alcoholic beverages. (Walker and Stewart, 2016). Acetaldehyde is produced in the initial step by removing the carboxyl group from pyruvate. Ethanol and acetic acid are then formed via the activities of alcohol dehydrogenase and aldehyde dehydrogenase, respectively. (Orywal and Szmitkowski, 2017). Ethyl acetate can also be generated from the combination of ethanol and acetic acid (Jørgensen et al., 2007). 2,3-Butanediol is mainly produced by bacterial species during sugar fermentation, being converted from 2,3-butanedione (diacetyl) and 3-hydroxy-2-butanone (acetoin). Pyruvate from glycolysis is condensed into α-acetolactate by α-acetolactate synthase (ALS), with α-acetolactate then being anaerobically transformed into acetoin by α-acetolactate decarboxylase (ALDC). Also, α-acetolactate can be decarboxylated to diacetyl and also reduced to 2,3-butanediol by 2,3-butanediol dehydrogenase (BDH) (Yang et al., 2017).

The fermentation of organic acids (e.g., propionic acid and butanoic acid) is mainly observed in the fermentation of certain microorganisms, such as *Propionibacterium acidipropionici* (Coral et al., 2008)*, Clostridium, Butyrivibrio, Eubacterium* and *Fusobacterium* species (Gonzalez-Garcia et al., 2017). The production of butanoic acid starts with the metabolism of glucose to pyruvate via the Embden–Mayerhof–Parnas (EMP) pathway, followed by conversion to acetyl-CoA, and then to acetoacetyl-CoA through a thiolase reaction. The butyryl-CoA produced from acetoacetyl-CoA is then converted into butyryl phosphate by phosphotransbutyrylase, followed by conversion into butanoate by butyrate kinase (Zhu and Yang, 2004). The metabolic pathways related to the production of propanoic acid can be divided into three classes: (i) acrylate and the Wood-Werkman-cycle pathway, (ii) the catabolism of amino acid pathways, and (iii) anabolic pathways associated with the production of biomass precursors from pyruvate or carbon dioxide (Gonzalez-Garcia et al., 2017).

**Fig. 3:**
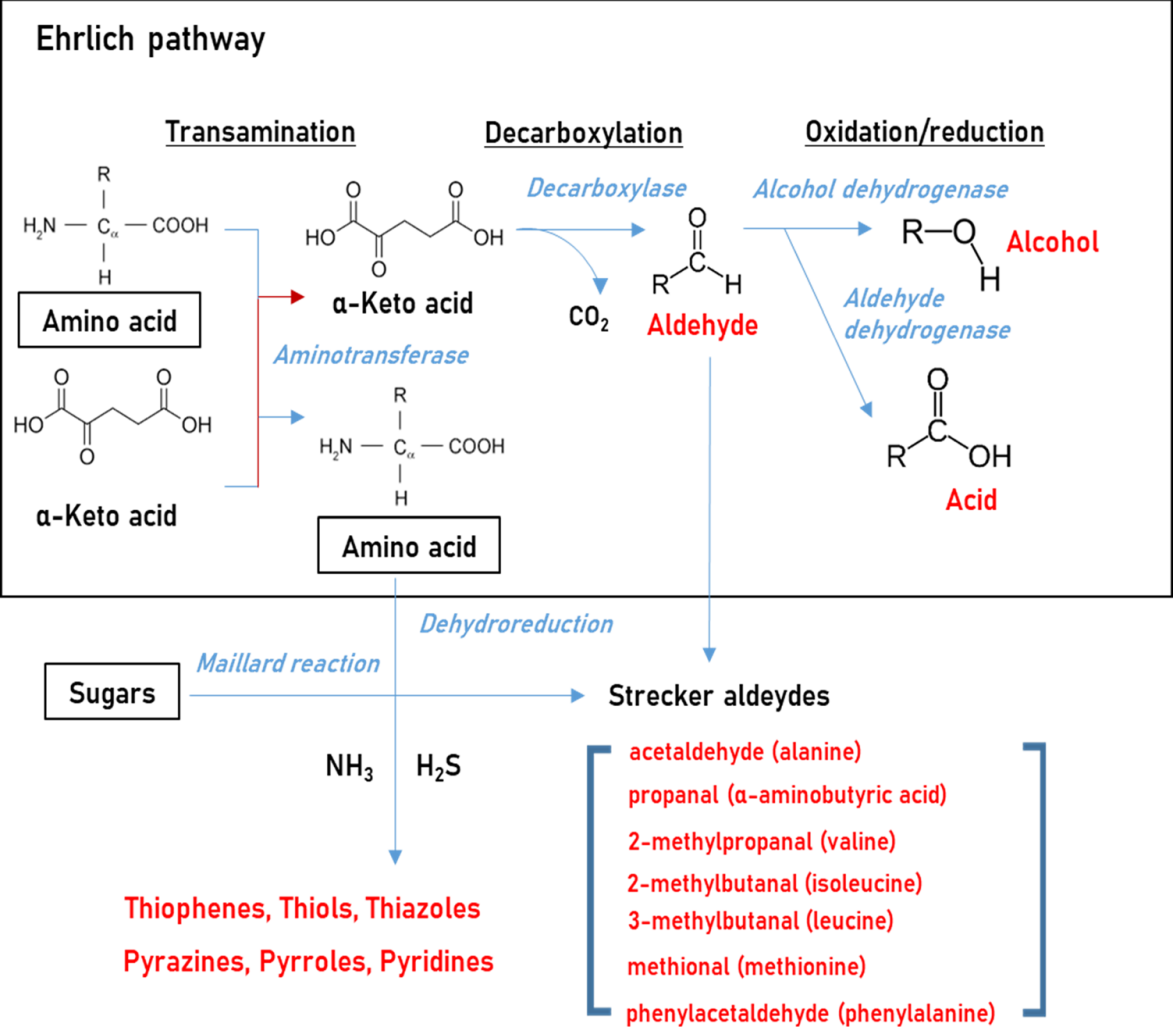
Scheme of Ehrlich pathway and amino acid degradation

Amino acids are common precursors involved in the production of higher alcohols (fusel alcohols) as well as sulfur- and nitrogen-containing volatile metabolites. The Ehrlich pathway is the main biological catabolism process involving branched-chain amino acids (leucine, isoleucine, and valine), aromatic amino acids (phenylalanine, tyrosine, and tryptophan), and sulfur-containing amino acids (cysteine and methionine), leading to the formation of fusel aldehydes, alcohols, acids, and esters. The Ehrlich pathway comprises three stages: (i) transamination, (ii) decarboxylation, and (iii) oxidation/reduction (Fig. 3). In the initial step, the amino group of amino acids is removed, transferred, or converted into ammonium ions by aminotransferase, and the remaining part participates in further reactions. Key intermediates from the Ehrlich pathway are α-keto acids, which are derived either catabolically from exogenous amino acids or result from anabolic pathways involving the biosynthesis of amino acids (Platell et al., 2000). Pyruvate decarboxylase is then converted into the resulting α-keto acids to aldehydes by decarboxylation. Finally, aldehydes are converted into acids and alcohols by alcohol dehydrogenase and acid dehydrogenase, respectively (Hazelwood et al., 2008).

Short-chain (comprising fewer than four carbon atoms) aliphatic aldehydes, acids, alcohols, and esters can be generated by the degradation of amino acids via the Ehrlich pathway as well as the degradation of fatty acids. (Hazelwood et al., 2008). This process involves generating various volatile aroma compounds, including carbonyls, higher alcohols, esters, volatile fatty acids, and sulfur compounds, and they are directly dependent on the nitrogen sources that are present during fermentation (Stribny et al., 2015; Torrea et al., 2011). Some volatile metabolites derived from amino acids play important roles in determining the sensory characteristics of fermented foods (Ardö, 2006; Etschmann et al., 2002; Park et al., 2007). In particular, branched-chain amino acids (leucine, isoleucine, and valine) are converted into corresponding aldehydes and acids with characteristic odor descriptions. For example, 3-methylbutanoic acid, which is derived from the degradation of leucine, has a low threshold value and distinctive odor notes (sweaty and cheese-like) and is mainly responsible for the off-odor note of *Cheonggukjang* (Korean traditional fermented soybeans) (Park et al., 2007). Methanethiol is released directly from the side group of methionine and further converted into other sulfur-containing compounds (Koval, 2005), and it may react with carboxyl acids to produce thioesters, which have boiled-cabbage- and cauliflower-like odor notes and are characteristic components of surface-ripened cheeses. (Ardö, 2006). On the other hand, aromatic amino acids (phenylalanine, tyrosine, and tryptophan) are converted into volatile metabolites contributing to flavors, such as rose-like, flowers, and bitter almond (e.g. benzaldehyde, 2-phenylethanol, and styrene) as well as chemical, putrid, and faecal flavors (e.g. indole and toluene) (Bertuzzi et al., 2018).

During fermentation, lipids can be hydrolyzed to glycerol and fatty acids by lipase. Glycerol can serve as a fermentation substrate for yeast as well as bacteria species (Wang et al., 2001). Some previous studies have shown that glycerol is also used as a carbon and energy source during carbohydrate metabolism (Himmi et al., 2000). The synthesized fatty acids comprise similar-length and straight carbon chains that have various numbers of carbon atoms (< C_6_, short chain; C_6–12_, medium chain; > C_14_, long chain) and different degrees of unsaturation (saturated, mono-unsaturated, and polyunsaturated) (Yu et al., 2014). They are usually degraded by β-oxidation, which is the breakdown of fatty acids into two carbon segments (acetyl-CoA) (Campbell et al., 2003). The degradation of fatty acids can generate further various volatile metabolites.

**Fig. 4:**
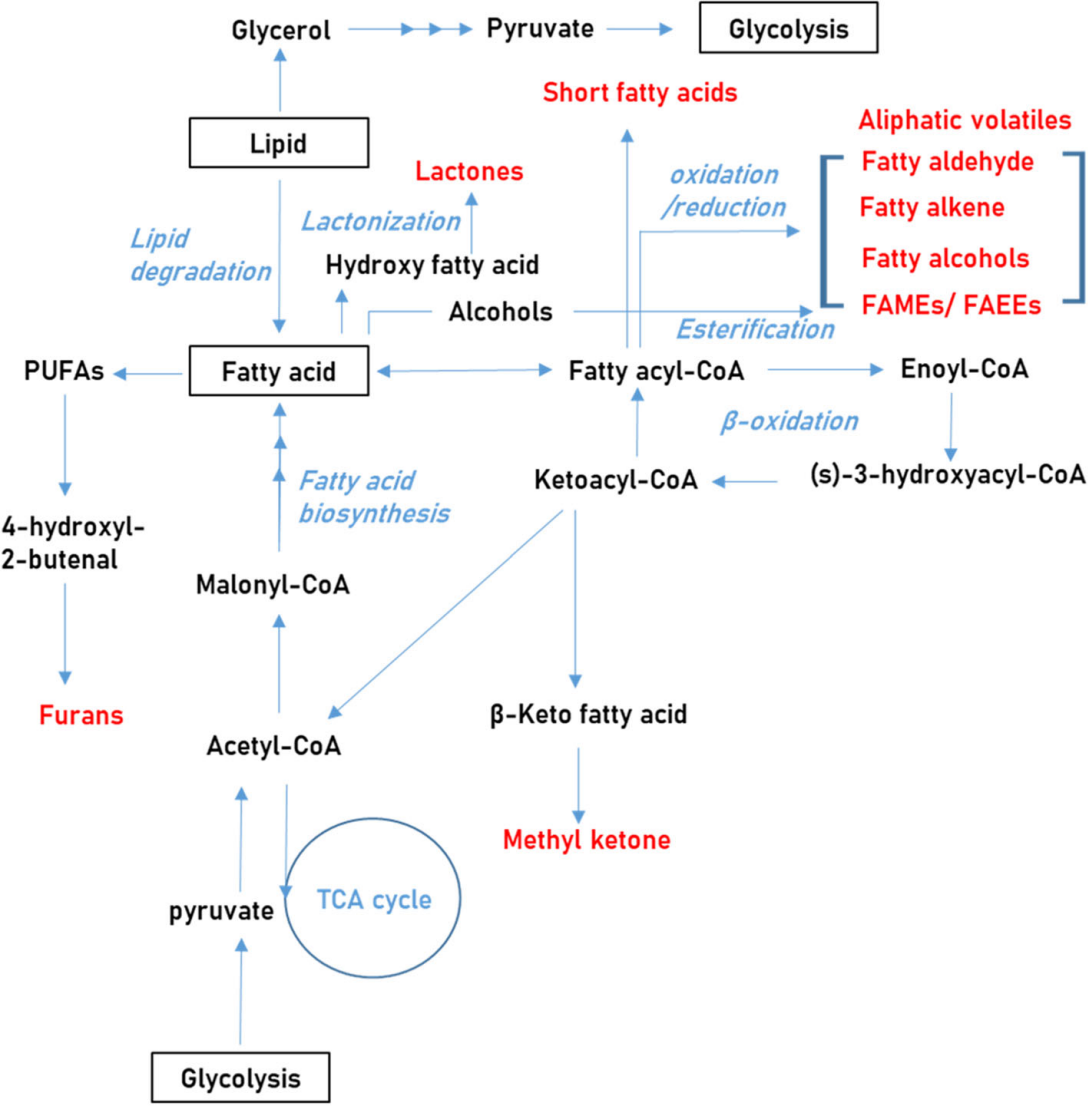
Overview of metabolic pathways that lead to production of fatty acids and fatty acid-derived metabolites. The abbreviations are shown as below; polyunsaturated fatty acids (PUFAs). fatty acid methyl esters (FAMEs), and fatty acid ethyl esters (FAEEs)

Figure 4 shows various fatty acids derived metabolites, such as fatty acid esters, straight chain aliphatic volatile metabolites (> C_6_), and lactones. Fatty acids-derived volatiles are mainly generated from β-oxidation or lipoxygenation of fatty acids (Catalá, 2010). Esters contribute to aroma properties such as fruity and flora odor descriptions in most fermented products (Francis and Newton, 2005; Reineccius. 2006), while esters with high molecular weights commonly exhibit fatty and oily aroma notes (Reineccius, 2006). Esters can be produced by the esterification of alcohols with fatty acids (Zhao et al., 2009).

Furans are mainly formed by the oxidative degradation of polyunsaturated fatty acids (Perez and Yaylayan, 2004). Some furan derivatives reportedly have characteristic odor descriptions. For example, furan compounds such as 2-pentylfuran, 2,3-dihydro-benzofuran, and 2-N-octylfuran are characterized by strongly scented, sweet, and (in some cases) burnt odor notes, and are generally considered to be important aromatic compounds that contribute greatly to the flavors of soy sauce (Diez-Simon et al., 2020). Lactones are produced by unsaturated fatty acids such as linoleic acid and oleic acid during fermentation. (Romero-Guido et al., 2011). These lactones have characteristic odor descriptions, such as buttery and peach-like odor notes, and contribute significantly to the aroma of certain fermented alcoholic beverages and dairy products (Zu and Xiao, 2019).

## Further omics strategies

An integrated omics approach has recently been regarded as an useful strategy for evaluating biological and functional properties at multiple scales to detect novel and hidden metabolites or pathways. Integrated omics data include multiple data sets collected using different technologies, such as genome-sequencing and metabolite profiles. Many possible pathways link genotypes and phenotypes, being represented by distinct functional states of cellular components (genes, metabolites, DNA methylation states, and proteins) (Fondi and Liò, 2015). Rossouw et al. (2012) used a multi-omics analysis to investigate the effects of the co-inoculation of commercial yeast (Saccharomyces cerevisiae) as a bacterial starter culture (*Oenococcus oeni*) in synthetic must. They compared transcriptome and flavor-active metabolite profiles, and some of the differentially expressed genes appeared to respond to chemical changes during fermentation. The up- and down-regulated genes were also determined, and the impacts on fermentation kinetics associated with the produced flavors and aromas were explained. Afshari et al. (2020) investigated the condition-specific microbes and metabolites on the surface of cheese using a multi-omics strategy (transcriptomics and metabolomics). They demonstrated that such a strategy represents a highly sensitive and reliable tool for distinguishing between closely related industrial and artisanal cheddar cheeses and brands (Afshari et al., 2020).

In general, the connection between gene expression and produced metabolites is estimated based on hypothesized cause-and-effect relationships. However, this approach can be challenging when interpreting data obtained using different technologies. Fondi and Liò (2015) proposed three main computational challenges: (i) tracking the different molecular components (i.e., genes, molecules, and enzymes) across different data sets and experiments, (ii) identifying (or developing) reliable data normalization procedures and multi-level data analysis, and (iii) producing an effective visualization of the results. These challenges—from the simple integration of two different data sets to integrating huge numbers of data sets—can be handled for obtaining holistic and comprehensive information about a specific phenomenon or microbial life as a whole. Various databases for metabolic systems are already available for many fermentative microorganisms, and software pipelines have been developed to create models that can predict outcomes when multi-omics data are imputed. (O'Donnell et al., 2020). This situation means that metabolomics now represents a very powerful tool for investigating the complex molecular and biological systems present in fermented foods.
